# Recovery of visual field defects following vitrectomy for proliferative diabetic retinopathy: A case report

**DOI:** 10.1097/MD.0000000000027793

**Published:** 2021-12-03

**Authors:** Kang-Jung Lo, Jin-Han Yang, Hui-Chen Cheng, Hsin-Yi Chang, Tai-Chi Lin

**Affiliations:** a Department of Ophthalmology, Taipei Veterans General Hospital, Taipei, Taiwan; b Department of Medical Education and Research, Taipei Veterans General Hospital, Taipei, Taiwan; c School of Medicine, National Yang Ming Chiao Tung University, Taipei, Taiwan; d Program in Molecular Medicine, School of Life Sciences, National Yang Ming Chiao Tung University, Taipei, Taiwan; e Department of Life Sciences and Institute of Genome Sciences, School of Life Sciences, National Yang Ming Chiao Tung University, Taipei, Taiwan; f Department of Ophthalmology, Shin Kong Wu Ho-Su Memorial Hospital, Taipei, Taiwan.

**Keywords:** proliferative diabetic retinopathy, visual field defect, vitrectomy

## Abstract

**Rationale:**

Proliferative diabetic retinopathy (PDR) may lead to severe visual impairment, and visual field (VF) loss in such patients has been reported. Vitrectomy is performed in PDR cases complicated with either vitreous hemorrhage or tractional retinal detachment to restore their visual acuity. However, its effect on VF defects is limited in data. Herein, we report the recovery of VF defects following vitrectomy in a patient with PDR.

**Patient concerns:**

A 25-year-old female with bilateral PDR and vitreous hemorrhage received 2 monthly intravitreal injections of aflibercept in both eyes. Six months after her last injection, she presented with fibrovascular membrane formation in both eyes and VF defects of −9.02 dB and −20.05 dB in the right and left eye, respectively.

**Diagnoses:**

Proliferative diabetic retinopathy in both eyes.

**Interventions:**

The patient underwent vitrectomy for her left eye.

**Outcomes:**

Although her visual acuity did not improve as expected, results from the Humphrey visual field analyzer showed notably improvement of her left eye (−9.05 dB) after the surgery.

**Lessons:**

Vitrectomy potentially allows recovery of VF defects in patients with PDR.

## 1 Introduction

Diabetic retinopathy (DR) is a disease with increasing prevalence which causes visual loss among working-age population in developed countries.^[1–3]^ Previous studies have documented visual field (VF) defects associated with DR.^[4,5]^ Vitrectomy potentially allows restoration of visual acuity in patients with proliferative diabetic retinopathy (PDR).^[6]^ However, changes in VF preoperatively and postoperatively is not routinely examined, and VF defects following vitrectomy has been of little discussion. Barsam reported that 2 thirds of patients may suffer from extensive VF loss after vitrectomy.^[7]^ Herein, we presented a case of recovery of VF defects following vitrectomy for PDR.

## 2 Case report

**Figure 1:**
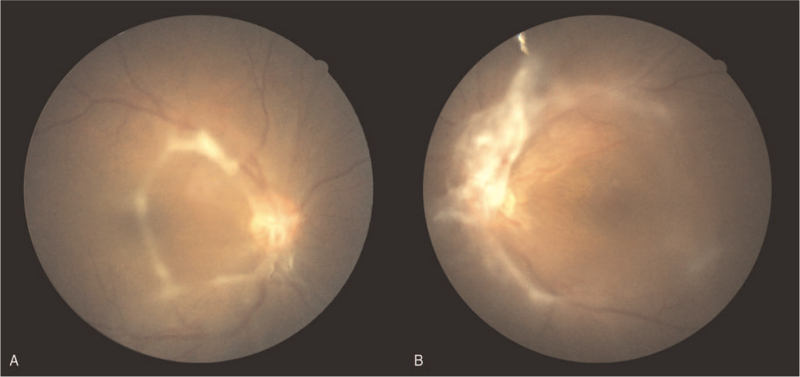
Fundoscopic examination revealed prominent fibrovascular tissue of (A) right eye and (B) left eye.

**Figure 2:**
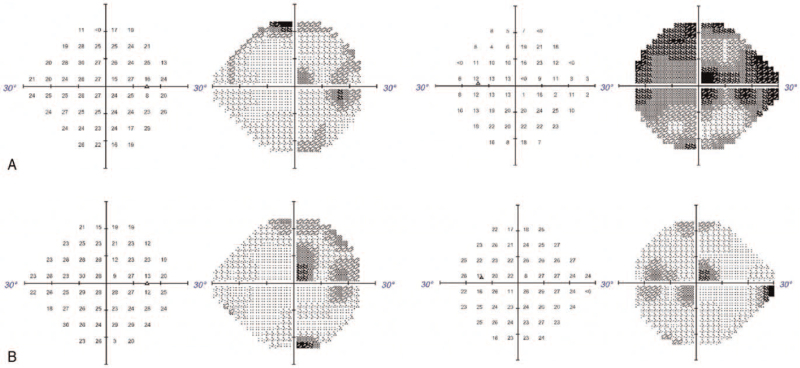
Humphrey visual field examination. (A) Before vitrectomy, the mean deviation of left eye was - 20.05 dB. The reduced visual sensitivity was more severe over peripheral area. (B) After vitrectomy, visual field showed notably improvement. The mean deviation of the left eye was - 9.05 dB.

A 25-year-old female with bilateral PDR and vitreous hemorrhage presented with decreasing best corrected visual acuity (BCVA) to counting finger at 50 cm in the right eye (OD), and 6/20 in the left eye (OS). She received 2 monthly intravitreal injections of aflibercept (2 mg/0.05 mL) in both eyes. After the first injection, vitreous hemorrhage subsided gradually and her BCVA improved to 6/12 OD and remained 6/20 OS. However, she complained narrowing of VF in the left eye 6 months after the second intravitreal injection. Fundoscopic exam showed fibrovascular membrane formation in both eyes (Fig. 1A, B). Monocular central 24 to 2 threshold test using a Humphrey visual field analyzer with the SITA-fast strategy showed mean deviation of −9.02 dB OD, and −20.05 dB OS (Fig. 2A). Optical Coherence Tomography revealed no diabetic macular edema in both eyes. Twenty three gauge vitrectomy and peeling of the fibrovascular membrane along with the internal limiting membrane was performed in the left eye. Two months after the surgery, her BCVA remained 6/20 OS. Significant improvement of mean deviation from −20.05 dB to −9.05 dB OS of the left eye was observed (Fig. 2B).

## 3 Discussion

It is essential for ophthalmologists to recognize the significance of visual impairment in patients with severe DR in terms of both visual acuity and VF. Vitrectomy is the surgery of choice to restore visual acuity in patients with PDR. However, its effect on recovery of VF defects in such patients is not clear.

It has been taken for granted that PDR and large hemorrhages could cause significant VF loss.^[4]^ Winznia et al reported that patient with DR would have VF defects associated with different diabetic lesions. For example, patients with macular hemorrhage and edema may suffer from central scotoma, and those with retinitis proliferans involving the optic disc would have arcuate scotomata.^[4]^ Bell and Feldon demonstrated that visual function determined by the Octopus automated static perimeter correlate linearly with capillary perfusion in 14 eyes with DR.^[8]^ Chee and Flanagan found that over 90% patients with capillary non-perfusion cause by DR suffered from significant VF loss.^[5]^ Furthermore, capillary non-perfusion in DR is closely associated with reduced retinal sensitivity.^[5]^

**Figure 3:**
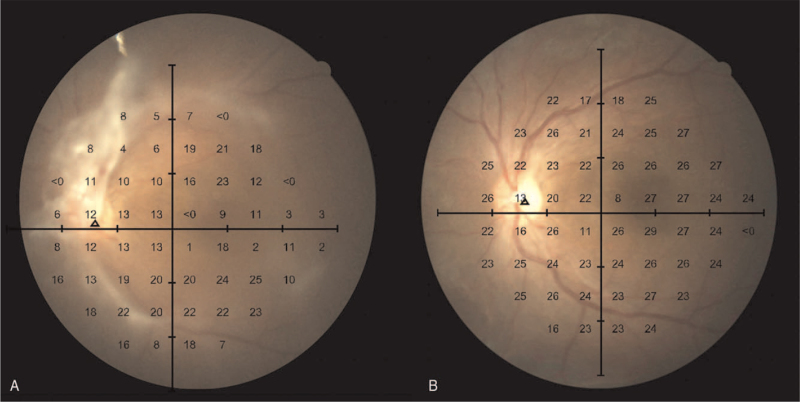
Humphrey 24 to 2 result superimposed onto inverted fundus color photos showing. (A) reduced retinal sensitivity before vitrectomy and (B) improved retinal sensitivity after vitrectomy.

In our case, the arcuate scotomata can be attributed to the formation of fibrovascular membrane connected to the optic disc. The central scotoma with areas of decreased retinal sensitivity is believed to be associated with capillary non-perfusion (Fig.3A). Sugiyama and Ando demonstrated the effect of vitrectomy on reperfusion of the capillary bed in eyes with DR.^[9]^ On the other hand, intravitreal aflibercept injection in diabetic macular edema patients has provided evidence of improving retinal perfusion associated with DR by decreasing retinal non-perfusion zone.^[10]^

Unlike previous study which reported that 2 thirds of patients may suffer from extensive VF loss after vitrectomy,^[7]^ our patient showed VF defects recovered following vitrectomy. The arcuate scotomata recovery may be due to removal of the fibrovascular membrane obstructing the perimetric stimulus light from reaching the retinal photoreceptors. The reclamation of central scotoma and retinal sensitivity advocates the reperfusion of the capillary bed (Fig. 3B). It is, however, difficult to determine the retinal perfusion status without performing fluorescein angiography prior to and following vitrectomy due to the retrospective nature of this case report.

In summary, our findings offer a rationale for the removing of the opaque media, and reperfusion of the capillary bed, which ultimately leads to the recovery of VF defects in patients with PDR following vitrectomy.

## Author contributions

**Writing – original draft:** Kang-Jung Lo, Jin-Han Yang, Hsin-Yi Chang.

**Writing – review & editing:** Hui-Chen Cheng, Tai-Chi Lin.
